# Dopaminergic System Alteration in Anxiety and Compulsive Disorders: A Systematic Review of Neuroimaging Studies

**DOI:** 10.3389/fnins.2020.608520

**Published:** 2020-12-03

**Authors:** Mei-Xue Dong, Guang-Hui Chen, Ling Hu

**Affiliations:** ^1^Department of Neurology, Hubei General Hospital, Renmin Hospital of Wuhan University, Wuhan, China; ^2^Department of Pharmacy, Hubei General Hospital, Renmin Hospital of Wuhan University, Wuhan, China

**Keywords:** anxiety, obsessive compulsive disorder, systematic review, dopamine, neuroimaging

## Abstract

**Objective:** The dopaminergic system is involved in many psychiatric disorders as a GABAergic, serotonergic, and glutamatergic system. A systematic review and meta-analysis was performed to elucidate the alteration of the dopaminergic system in anxiety and compulsive disorders.

**Methods:** The databases of Pubmed, Embase, and ScienceDirect were searched and articles reporting the involvement of the dopaminergic system in patients with anxiety disorder and obsessive compulsive disorder (OCD) were recognized. The key research data were extracted from the included articles and standardized mean differences were calculated using meta-analyses if there were more than two studies with obtainable data. Sensitivity analyses were further performed to detect the stability of results, and the qualities of all the included studies were assessed using the Newcastle Ottawa scale.

**Results:** Finally, we identified 8 and 11 studies associated with anxiety disorder and OCD for further analysis, respectively. Most consistently, the striatal dopamine D_2_ receptor (D_2_R) of OCD patients had decreased while no significant correlation was found between striatal D2R and disease severity. The striatal dopamine transporter (DAT) had not been significantly altered in both the anxiety disorder and OCD patients. The heterogeneity values from the meta-analyses were extremely high while those results remained stable after sensitivity analyses. Inconsistent data were found in the striatal D_2_R of patients with anxiety disorder. Limited data had suggested that dopamine synthesis increased in most regions of the cerebral cortex and cerebellum in OCD patients.

**Conclusions:** The most convincing finding was that the D_2_ receptor decreased in patients with obsessive compulsive disorder. The dopamine transporter may have no relationship with anxiety and compulsive disorder.

## Introduction

Anxiety and compulsive disorder has been recognized as one of the most prevalent psychiatric disorders in the Chinese mainland and its prevalence has ranged from 24.47 to 41.12% (Guo et al., 2016). This broad-spectrum disorder includes generalized anxiety disorder (GAD), non-specific anxiety disorder, panic disorder with or without agoraphobia, social anxiety disorder (SAD), specific phobia, post traumatic stress disorder (PTSD), and obsessive compulsive disorder (OCD) according to the former diagnostic criteria, while OCD and PTSD have been listed as independent diseases according to the recent Diagnostic and Statistical Manual of Mental Disorders, version V (DSM-V).

Various neurotransmitters have been implicated in the pathophysiology of anxiety and compulsive disorder including dopamine (Plavén Sigray et al., 2016), serotonin (Nikolaus et al., 2016), glutamate (Spencer et al., 2014), and γ-aminobutyric acid (GABA) (Mohler, 2012). A meta-analysis of ^1^H magnetic resonance spectroscopy (MRS) reported that no significant differences in GABA levels were found in panic disorder patients (Schür et al., 2016) while the GABA(A) receptor was reported to decrease throughout the mesolimbocortical system in anxiety disorder (Nikolaus et al., 2014). Another systematic review found that patients with anxiety-related disorder displayed a significant reduction in the serotonin transporter in the thalamus, amygdala, and hippocampus, reductions of 5-HT_1A_ receptors in the frontal cortex, cingulate, midbrain, hippocampus, amygdala, and insula, and a significant elevation of 5-HT_2A_ receptors in the temporal cortex (Nikolaus et al., 2016). The increase of striatal Glx (combination of glutamate, glutamine, and GABA) in OCD patients was also concluded in a review (Naaijen et al., 2015).

There is sufficient evidence for the involvement of the dopaminergic system in psychiatric disorders such as schizophrenia (Horga et al., 2016), major depression disorder (Wooten et al., 2015), bipolar disorder (Yatham et al., 2002), attention deficit hyperactive disorder (ADHD) (Badgaiyan, 2016), anorexia nervosa (Broft et al., 2015), Tourette syndrome (Steeves et al., 2010), and autism (Nakamura et al., 2010). Dopamine is an important neurotransmitter in the central nervous system and is known to regulate human emotions and cognitive abilities, including feeling, thinking, understanding, and reasoning in physiological processes (Pine et al., 2010). A variety of approaches have been applied to determine the role of the dopaminergic system in the brain, however, positron emission tomography (PET) and single photon emission computed tomography (SPECT) are the only methods used to explore the dopaminergic system directly *in vivo*. We can now assess dopamine synthesis, release, synaptic vesicular transporters, dopaminergic receptors, and DAT *in vivo* using PET or SPECT with a radiotracer such as [^18^F]-DOPA, [^11^C]-DOPA, [^11^C]-DTBZ, [^123^I]-FP-CIT, [^123^I]-β-CIT, [^11^C]-PE2I, [^11^C]-CFT, [^99m^Tc]-TRODAT, [^11^C]-SCH 23390, [^11^C]-NNC 112, [^123^I]-IBZM, [^11^C]-RAC, and [^18^F]-fallypride (Fusar-Poli and Meyer-Lindenberg, 2013). Taking advantage of these examinations, dopaminergic system alteration has been gradually cleared in many psychiatric disorders such as schizophrenia (Fusar-Poli and Meyer-Lindenberg, 2013), ADHD (Fusar-Poli et al., 2012), and major depression disorder (Li et al., 2015).

We have performed a number of studies on affective diseases and Parkinsonism-related disorders and found that the dopaminergic system might play an important role in anxiety disorders (Dong et al., 2018a,b). Nearly half of patients with Parkinson's disease (PD) suffer serious anxiety disorders while the dopaminergic system is confirmed to be the main pathological footstone of PD. Dopamine agonist pramipexole was also reported to alleviate mood disorder in PD patients. There are a number of studies that have focused on the dopaminergic system in anxiety and compulsive disorder but the results were inconsistent with each other. The lack of conformity and fragmentary nature of these results prompted us to reassess the dopaminergic system in patients with anxiety and compulsive disorder. Thus, we performed this systematic review and meta-analysis to elucidate dopaminergic system alteration in anxiety and compulsive disorder.

## Methods

We carried out the systematic review and meta-analysis following the guidelines recommended by PRISMA statements and the protocol that has already been registered with PROSPERO (CRD42016046788) (Moher et al., 2010).

### Data Sources and Searches

Pubmed, Embase, and ScienceDirect were searched without restrictions of language, publication type, or publication period. The detailed search strategy is shown in Table 1. Briefly, our search criteria included articles that had words related to each of the following three categories in the title or abstract: (i) anxiety-related disorder or OCD; (ii) DAT, dopamine receptor, dopamine synthesis, or dopamine release; (iii) PET or SPECT *in vivo* studies. This search retrieved all articles through to August 20, 2020. In addition, a backward search of bibliographic references from the identified articles was performed and articles were examined to verify that all relevant articles were included in this article.

**Table 1:** Search strategy of database.

**Data source**	**Search strategy**	**Total count**
Pubmed	(((((((((((((anxiety[Title/Abstract]) OR phobia[Title/Abstract]) OR panic[Title/Abstract]) OR agoraphobia[Title/Abstract]) OR ptsd[Title/Abstract]) OR gad[Title/Abstract]) OR ocd[Title/Abstract]) OR obsessive compulsive disorder[Title/Abstract]) OR compulsive[Title/Abstract]) OR impulsive[Title/Abstract])) AND (((dopamine[Title/Abstract]) OR dopaminergic[Title/Abstract]) OR daergic[Title/Abstract])) AND ((((((spect[Title/Abstract]) OR spet[Title/Abstract]) OR pet[Title/Abstract]) OR positron emission tomography[Title/Abstract]) OR single photon emission computed tomography[Title/Abstract]) OR single photon emission tomography[Title/Abstract])	255
Embase	“anxiety”:ab OR “phobia”:ab OR “panic”:ab OR “agoraphobia”:ab OR “gad”:ab OR “ocd”:ab OR “obsessive compulsive disorder”:ab OR “compulsive”:ab OR "impulsive”:ab AND (“spect”:ab OR “spet”:ab OR “pet”:ab OR “single photon emission computed tomography”:ab OR “positron emission tomography”:ab OR “single photon emission tomography”:ab) AND (“dopamine”:ab OR “dopaminergic”:ab OR “daergic”:ab)	446
ScienceDirect	[abstract (anxiety) or abstract (phobia) or abstract (panic) or abstract (agoraphobia) or abstract (gad) or abstract (ocd) or abstract (obsessive compulsive disorder) or abstract (compulsive) or abstract (impulsive)] and [abstract (spect) or abstract (pet) or abstract (spet) or abstract (single photon emission computed tomography) or abstract (positron emission tomography)] and [abstract (dopamine) or abstract (dopaminergic) or abstract (daergic)]	79

### Study Selection

Each article obtained from the search strategy was then reviewed to determine its inclusion or exclusion according to the following criteria. The included articles should compare related psychiatric patients with healthy controls. In the meanwhile, dopaminergic systems, including dopamine synthesis, dopamine release, dopaminergic receptors, or DAT, should be determined by PET or SPECT *in vivo* between the two groups. The exclusion criteria were reviews, case reports, protocols, animal studies, repeated reports from the same research group, and any other research purposes.

### Data Extraction

Finally, the included articles were reviewed by two skillful reviewers devoted to psychiatric disorders (MXD and LH). We recorded the following variables from each article: article information, characteristics of subjects, diagnostic criteria, disease severity scale score, assessment method, reference region, region of interest (ROI), and dopaminergic findings. A third reviewer (GHC) then reviewed all the articles and extracted data to check for any errors. Information regarding the direction and significance of the dopaminergic system (including mean values and standard deviation values) of ROI were then used for further analysis.

### Statistical Methods

A meta-analysis was conducted to merge the effect size together if there were more than two studies with obtainable data. The process of statistical analysis was as described before (Dong et al., 2016). Briefly, standardized mean differences (SMDs) were calculated to assess the changes of each efficacy outcome for continuous measures and combined into a pooled SMD. If data were measured several times in different parts of the same brain region in one individual, a weighted average and standard deviation were calculated according to the following formulas $\text{merged}\;\text{N}=\frac{{\text{N}}_{1}+{\text{N}}_{2}}{2}$, $\text{merged mean}=\frac{{\text{N}}_{1}{\text{M}}_{1}+{\text{N}}_{2}{\text{M}}_{2}}{{\text{N}}_{1}+{\text{N}}_{2}}$, and merged standard deviation $=\sqrt{\begin{array}{l} (\left({\text{N}}_{1}-\text{1}\right){\text{SD}}_{1}^{2}+\left({\text{N}}_{2}-\text{1}\right){\text{SD}}_{2}^{2}\\ +\frac{N_{1}N_{2}}{\left(N_{1}+N_{2}\right)\left(M_{1}^{2}+M_{2}^{2}-2M_{1}M_{2}\right)}))/(N_{1}+N_{2}-1) \end{array}}$. Heterogeneity across studies was assessed using Chi^2^ test and expressed using *I*^2^ statistic values. An *I*^2^ of <25%, <50%, <75%, and ≥75% represented low, moderate, high, and extremely high heterogeneity, respectively. Sensitivity analyses were conducted using the leave-one-out method. Data were analyzed using RevMan5.4 (Cochrane Information Management System).

## Results

### Literature Search Results

**Figure 1:**
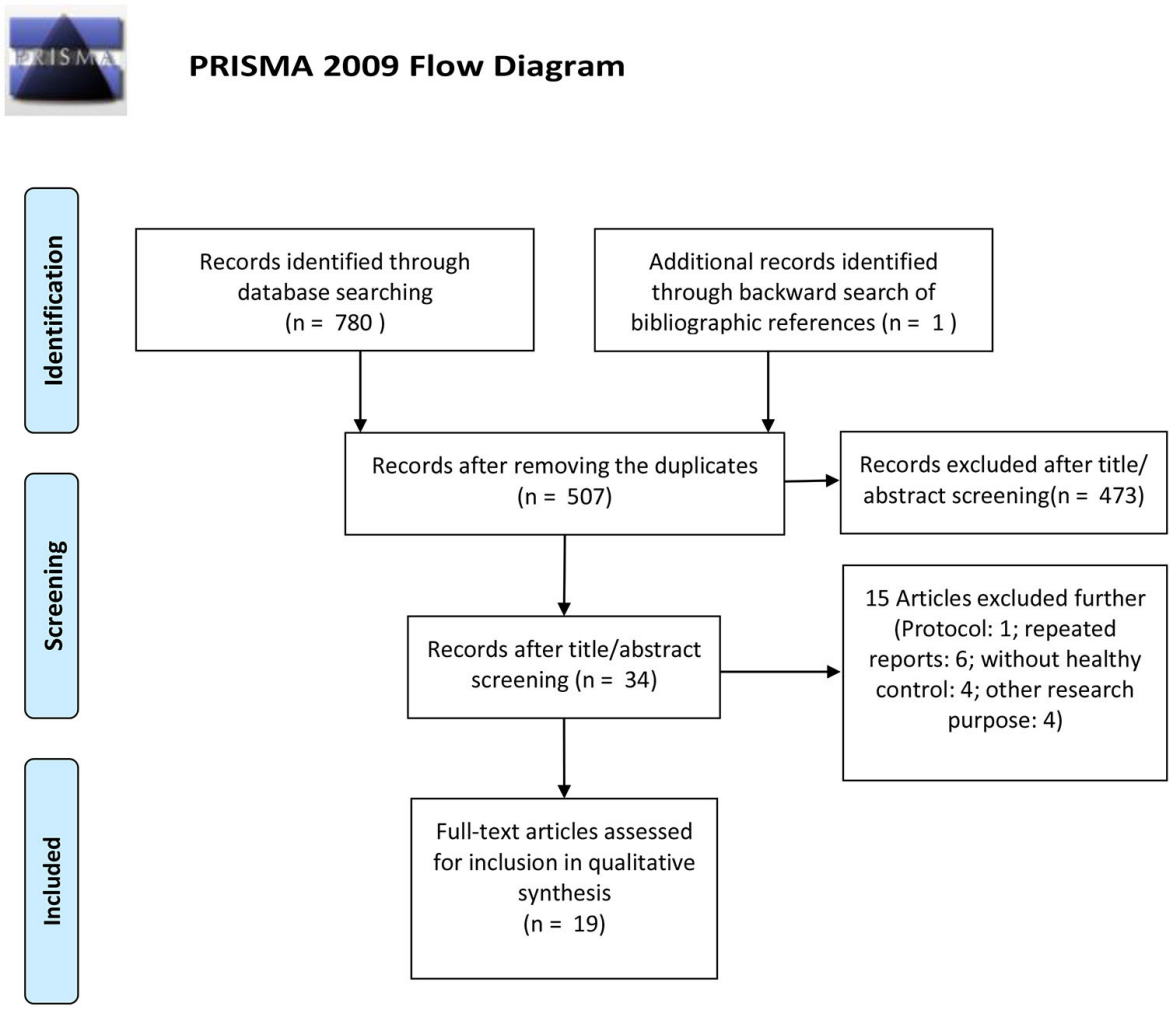
PRISMA flow diagram of this systematic review and meta-analysis.

The detailed flowchart of the study selection is shown in Figure 1. A total of 781 records were initially identified and 507 records were left after duplicate records were removed; of these, 473 records were then excluded by title/abstract screening. Of the 34 remaining relevant records, 15 records were further excluded as they were protocol reports, repeated reports from the same research team, a study without healthy controls, or had another research purpose. Thus, 19 studies were finally included in this review (Tiihonen et al., 1997; Schneier et al., 2000, 2008, 2009; Kim et al., 2003; Denys et al., 2004, 2013; Van der Wee et al., 2004, 2008; Hesse et al., 2005; Perani et al., 2008; Olver et al., 2009, 2010; Maron et al., 2010; Hoexter et al., 2013; Hsieh et al., 2014; Lee et al., 2015; Plavén-Sigray et al., 2017; Hjorth et al., 2019). We then aggregated these studies by psychiatric disorders, including anxiety disorder and obsessive compulsive disorder. The characteristics of each study are exhibited in Table 2. The Newcastle Ottawa scales of all the included studies are shown in Table 3.

**Table 2:** Studies assessing dopaminergic system using PET or SPECT in patient with anxiety and compulsive disorder.

**Study**	**Subjects**	**Diagnostic criteria**	**Method**	**RR**	**ROI**	**Dopaminergic system findings**
**Anxiety disorder (8)**						
Tiihonen et al. (1997) Finland	11 SAD (age 40.5 ± 5.3, 3 males); 28 controls (age 39.6 ± 12.3, 19 males)	DSM-III-R SAD; CGI 5.09 ± 0.54; Drug free	SPECT, [^123^I]-β-CIT	White matter	Striatum (basal anglia)	DAT decreased in striatum (10.05 ± 1.20 vs. 11.64 ± 1.39, *p* < 0.001); No significant correlation between disease severity and striatal DAT
Van der Wee et al. (2008) Netherlands	12 SAD (age 39.4 ± 12.6, 7 males); 12 controls (age 33.0 ± 9.5, 7 males)	DSM-IV SAD; LSAS 73.6 ± 13.7; Drug naïve	SPECT, [^123^I]-β-CIT	Cerebellum	Striatum	DAT increased in striatum (7.30 ± 0.98 vs. 5.47 ± 1.37, *p* = 0.011); No significant correlation between LSAS score and striatal DAT
Maron et al. (2010) Estonia	7 current PD (35.7 ± 16.8, 0 male); 7 PD in remission (33.4 ± 14.3, 0 male); 7 controls (35.1 ± 5.3, 0 male)	DSM-IV PD; PDSS (8.9 ± 2.3); Drug free (>4 m)	SPECT, [^123^I]-β-CIT	Cerebellum	Striatum	DAT increased in striatum of remitted PD (2.72 ± 0.27, *p* = 0.04), didn't change in striatum of current PD compared with control (2.21 ± 0.29 vs. 2.43 ± 0.17, *p* = 0.12); There was an inverse relationship between striatal DAT in total group and the PDSS score, no correlation exist between these two subgroups
Lee et al. (2015) China	12 GAD (age 37.1 ± 12.6, 4 males); 12 controls (age 37.2 ± 12.9, 4 males)	DSM-IV GAD; HAM-A; Drug free (>3 m)	SPECT, [^99m^Tc]-TRODAT-1	Occipital cortex	Striatum	DAT decreased in striatum (1.53 ± 0.46 vs. 2.01 ± 0.31, *p* = 0.004)
Hjorth et al. (2019) Sweden	27 SAD (age 31.1 ± 10.32, 17 males); 43 controls (age 32.81 ± 11.56, 23 males)	DSM-IV SAD; LSAS-SR 84.96 ± 20.37; Drug free (>3 m)	PET, [^11^C]-PE2I	Cerebellar gray matter	Amygdala, hippocampus, caudate, putamen	DAT increased in amygdala, hippocampus, caudate, and putamen; There was a positive correlation between disease severity and DAT availability in the amygdala, hippocampus, and putamen
Schneier et al. (2009) America	15 SAD (age 31.1 ± 6.6); 12 controls (age 30.9 ± 8.1)	DSM-IV SAD; LSAS 78.6 ± 19.2; Drug free (several months)	SPECT, [^123^I]-β-CIT; PET, [^11^C]-RAC;	Occipital region	Striatum	DAT did not differ in striatum (7.69 ± 1.12 vs. 7.62 ± 0.91, *p* = 0.87), D_2_R did not differ at baseline (13.0 ± 3.7 vs. 13.8 ± 3.2, *p* = 0.58); No significant correlation between LSAS score and striatal DAT, D_2_R and dopamine release
Schneier et al. (2000) America	10 SAD (age 32.5 ± 10.4, 5 males); 10 controls (age matched)	DSM-IV SAD; LSAS; Drug free (>1 y)	SPCET, [^123^I]-IBZM	Occipital region	Striatum	D_2_R decreased in striatum (93.6 ± 29.8 vs. 133.5 ± 38.2, *p* = 0.02); No significant correlation between LSAS score and striatal D_2_R
Plavén-Sigray et al. (2017) Sweden	12 SAD (age 33.82 ± 11.55); 16 controls (age 37.82 ± 15.22)	DSM-IV SAD; LSAS-SR 63.73 ± 10.48; Drug free	PET, [^11^C]-FLB457	Cerebellum	Lateral prefrontal cortex, medial frontal cortex, orbitofrontal cortex, anterior cingulated cortex, insula, amygdala, hippocampus	D_2_R increased in the orbitofrontal cortex (0.99 ± 0.28 vs. 0.76 ± 0.27, *p* = 0.03), no significant differences in the other regions; There was a positive correlation between LSAS-SR summed scores and orbitofrontal cortex D_2_R
**Obsessive compulsive disorder (11)**						
Kim et al. (2003) South Korea	15 OCD (age 28.53 ± 10.91, 11 males); 19 controls (age 30.53 ± 8.82, 11 males)	DSM-IV-Korean version; Y-BOCS 15.27 ± 3.86; Drug free (>4 w)	SPECT, [^123^I]-IPT	Occipital cortex	Striatum (basal ganglia)	DAT increased in the right basal ganglia (6.84 ± 2.82 vs. 4.61 ± 1.06, p=0.009) while it tends to increase in the left basal ganglia (6.74 ± 3.36 vs. 4.85 ± 1.13, *p* = 0.06); No significant correlation between Y-BOCS score and striatal DAT
Van der Wee et al. (2004) Netherlands	15 OCD (age 31.4 ± 9.0, 11 males); 15 controls (age 32.0 ± 9.5, 11 males)	DSM-IV; Y-BOCS 23.4 ± 4.2; Drug naïve	SPECT, [^123^I]-β-CIT	Cerebellum	Striatum (caudate, putamen)	DAT increased in left caudate (6.80 ± 0.64 vs. 5.99 ± 0.78, *p* = 0.004), right caudate (6.78 ± 0.67 vs. 6.16 ± 0.85, *p* = 0.04), left putamen (8.00 ± 0.74 vs. 7.04 ± 0.79, *p* = 0.006), and right putamen (7.97 ± 0.89 vs. 7.16 ± 1.23, *p* = 0.05); No significant correlation between DAT and Y-BOCS score
Hesse et al. (2005) Germany	15 OCD (age 32.1 ± 11.7, 8 males); 10 controls (40 ± 13.2, 7 males)	ICD-10; Y-BOCS (25.3 ± 8.8); Drug naïve	SPECT, [^123^I]-β-CIT	Occipital cortex	Striatum Thalamus Midbrain Brainstem	DAT decreased in striatum (13.2 ± 1.6 vs. 14.9 ± 3.3, *p* = 0.001), thalamus/hypothalamus (4.2 ± 0.9 vs. 4.9 ± 0.8, *p* = 0.026), midbrain (2.5 ± 0.6 vs. 3.3 ± 0.8, *p* = 0.008), and brainstem (1.7 ± 0.6 vs. 2.4 ± 0.7, *p* = 0.014); No significant correlation between DAT and Y-BOCS score
Hoexter et al. (2013) Brazil	41 OCD (age 30.6 ± 11.3); 32 controls (age matched)	DSM-IV; Y-BOCS; Drug naive	SPECT, [^99m^Tc]-TRODAT-1	Cerebellum	Striatum (anterior putamen)	DAT decreased in the right anterior putamen (2.05 ± 0.36 vs. 2.24 ± 0.37, *p* = 0.031) and a statistical tendency in the left anterior putamen (2.04 ± 0.40 vs. 2.27 ± 0.55, *p* = 0.071)
Olver et al. (2009) Australia	7 OCD (age 40.0 ± 13.9, 4 males); 7 controls (age 40.3 ± 12.3, 4 males)	DSM-IV; Y-BOCS 22.1 ± 7.6; Drug free (>10 d)	PET, [^11^C]-SCH23390	Cerebellum	Striatum	D_1_R decreased in caudate compared with controls (0.59 ± 0.06 vs. 0.88 ± 0.06, *p* < 0.05), and it also decreased in putamen (0.89 ± 0.06 vs. 1.14 ± 0.06, *p* < 0.05); No significant correlations between D_1_R and Y-BOCS score
Olver et al. (2010) Australia	7 OCD (age 40.0 ± 13.9, 4 males); 7 controls (age 40.3 ± 12.3, 4 males)	DSM-IV; Y-BOCS 22.1 ± 7.6; Drug free (>10 d)	PET, [^11^C]-SCH23390	Cerebellum	Anterior cingulate cortex	D_1_R decreased in left anterior cingulate compared with controls (0.14 ± 0.04 vs. 0.29 ± 0.01, *p* < 0.05), and it also decreased in the right (0.13 ± 0.03 vs. 0.25 ± 0.02, *p* < 0.05); High negative correlations were found between D_1_R score and Y-BOCS total score
Denys et al. (2004) Netherlands	10 OCD (age 36.4 ± 12, 3 males); 10 controls (age 33.7 ± 10, 3 males)	DSM-IV; Y-BOCS 25.9 ± 6.5; 2 drug naïve, 8 drug free (>1 m)	SPECT, [^123^I]-IBZM	Cerebellum	Striatum (caudate, putamen)	D_2_R decreased in left caudate nucleus (*p* = 0.016) while it decreased not significantly in right caudate, left and right putamen; No significant correlation between Y-BOCS score and D_2_R
Schneier et al. (2008) America	8 OCD (age 32.50 ± 12.15, 6 males); 7 controls (age 29.71 ± 10.73, 4 males)	DSM-IV; Y-BOCS 22.75 ± 6.09; Drug free (>2 m)	SPECT, [^123^I]-IBZM	Occipital cortex	Striatum	D_2_R did not differ significantly compared with controls (93.08 ± 36.90 vs. 118.41 ± 45.40, *p* = 0.247); No significant correlation between Y-BOCS score and striatal D_2_R
Perani et al. (2008) Italy	9 OCD (age 30.56 ± 6.93, 6 males); 9 controls (age 26.55 ± 8.32, 8 males)	DSM-IV; Y-BOCS 29.44 ± 4.42; Drug naïve	PET, [^11^C]-RAC	Cerebellum	Striatum (dorsal caudate, ventral basal ganglia)	D_2_R decreased in dorsal caudate (2.05 ± 0.191 vs. 2.28 ± 0.093, *p* = 0.0057), dorsal putamen (2.41 ± 0.213 vs. 2.68 ± 0.083, *p* = 0.0026), and ventral basal ganglia (1.57 ± 0.113 vs. 1.86 ± 0.098, *p* = 0.0000); No significant correlations between D_2_R and disease severity
Denys et al. (2013) Netherlands	12 OCD (age 35.8 ± 11.5, 4 males); 12 controls (age 32 ± 12, 4 males)	SCID-I; Y-BOCS (23.6 ± 5.5); 7 drug naïve, 5 drug free (>6 m)	PET, [^11^C]-RAC	Cerebellum	Striatum (putamen)	D_2_R decreased in left putamen (*p* < 0.005) and didn't change after amphetamine in striatum; No significant correlation between Y-BOCS score and striatal D_2_R or their changes
Hsieh et al. (2014) China	5 OCD (age 33.2 ± 11.2, 4 males); 6 controls (age 22.8 ± 1.2, 4 males)	DSM-IV; Y-BOCS (25.00 ± 7.25); 5 drug free (>5 d)	PET, [^18^F]-FDOPA	Not Given	Frontal premotor cortex, posterior cingulate gyrus, cuneus, precuneus, lingual gyrus, middle temporal gyrus, cerebellum in both hemispheres	Dopamine synthesis increased in left frontal premotor cortex, left posterior cingulate gyrus, left cuneus, left lingual gyrus, right cuneus and precuneus, right lingual gyrus, right middle temporal gyrus, left cerebellum, and right cerebellum (*p* < 0.01)

*PET, positron emission tomography; SPECT, single photon emission computed tomography; RR, reference region; ROI, region of interest; SAD, social anxiety disorder; DSM, Diagnostic and Statistical manual of Mental disorders; CGI, Clinical Global Impression scale; DAT, dopamine transporter; MRI, magnetic resonance imaging; LSAS, Liebowitz Social anxiety scale; GAD, generalized anxiety disorder; HAM-A, Hamilton Anxiety Rating Scale; D_2_R, dopamine D_2_ receptor; PD, panic disorder; PDSS, Panic Disorder Severity Scale; OCD, obsessive-compulsive disorder; D_1_R, dopamine D_1_ receptor; SCID-I, Structural Clinical Interview for DSM-IV axis I disorders; Y-BOCS, Yale-Brown Obsessive Compulsive Scale; ICD, International Classification of Disease*.

**Table 3:** Newcastle Ottawa scales of the included studies.

**Author**	**Year**	**Selection**				**Comparability**	**Exposure**		**Total**
		**1**	**2**	**3**	**4**		**1**	**2**	
Tiihonen	1997	1	0	0	1	2	0	1	5
Wee	2008	1	0	1	1	2	0	1	6
Schneier	2009	1	0	1	1	2	0	1	6
Schneier	2000	1	0	1	1	2	0	1	6
Sigray	2017	1	0	1	1	2	0	1	6
Lee	2015	1	0	1	1	2	0	1	6
Maron	2010	0	0	0	1	2	0	1	4
Kim	2003	1	0	0	1	1	0	1	4
Wee	2004	1	1	1	1	2	0	1	7
Hesse	2005	1	0	1	1	1	0	1	5
Hoexter	2013	0	0	0	1	1	0	1	3
Olver	2009	1	0	0	1	2	0	1	5
Olver	2010	1	0	0	1	2	0	1	5
Denys	2004	1	0	1	1	2	0	1	6
Schneier	2008	1	0	1	1	2	0	1	6
Perani	2008	1	1	1	1	1	0	1	6
Denys	2013	1	0	1	1	1	0	1	5
Hsieh	2014	1	0	0	1	2	0	1	5
Hjorth	2019	1	0	1	1	2	0	1	6

*Selection: 1, one score can be given if the case definition is adequate with independent validation; 2, one score can be given if the cases are consecutive or obviously representative series of cases; 3, one score can be given if the controls are community controls; 4, one score can be given if controls is without history of disease in the end of research. Comparability: one score can be given if controls are matched with age; two scores can be given if any additional factor are matched except age. Exposure: 1, one score can be given if PET or SPECT are assessed blindly for investigators; 2, one score can be given if same method of ascertainment is for cases and controls*.

### Dopaminergic System in Patients With Anxiety Disorder

**Figure 2:**
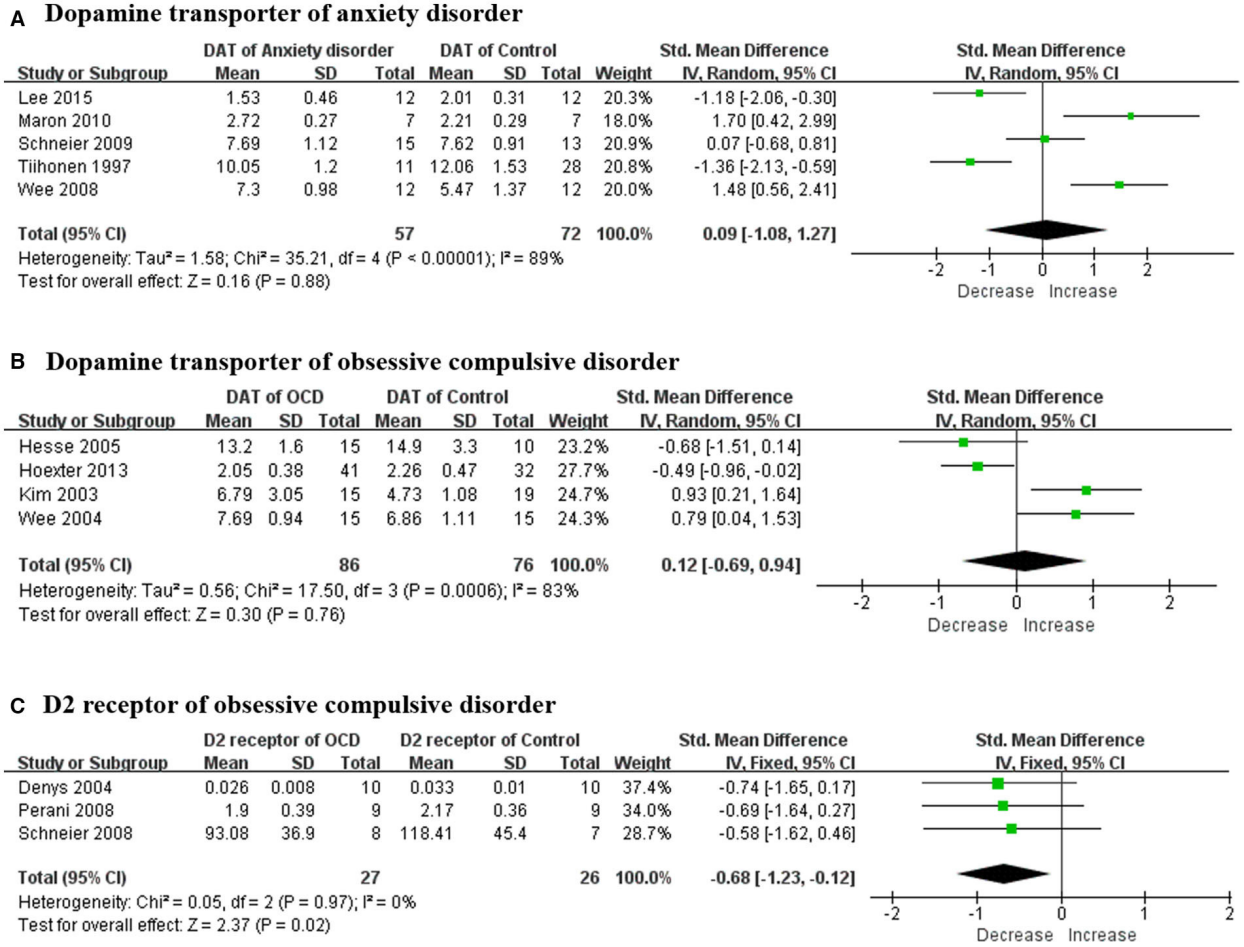
Forest plots of SMDs in the meta-analysis. **(A)** DAT had not altered in patients with anxiety disorder (95% CI of SMD, −1.49 to 1.59) and the heterogeneity was extremely high (*I*^2^ = 91%); **(B)** DAT had not altered in OCD patient (95% CI of SMD, −0.69 to 0.94) while the heterogeneity was extremely high (*I*^2^ = 83%); and **(C)** dopamine D_2_ receptors had decreased in OCD patient (95% CI of SMD, −1.23 to −0.12) and the heterogeneity was mild (*I*^2^ = 0%). SMD, standard mean deviation; DAT, dopamine transporter; OCD, obsessive compulsive disorder.

There were a total of eight studies focusing on the dopaminergic system in patients with anxiety disorder. The mean age of subjects ranged from 30 to 40 years old without statistically significant differences, and no specific pattern of age emerged among these studies. Striatal DAT was reported in six studies and the results were different from each other. A meta-analysis was conducted and no statistically positive effect was found as the total pooled SMD was 0.09 [95% confidence interval (CI), −1.08 to 1.27] (Figure 2A). The heterogeneity was extremely high (*I*^2^ = 89%) while the result remained stable through sensitivity analyses (Table 4). All of them reported no significant correlation between striatal DAT and disease severity. Only one study (Hjorth et al., 2019) assessed an extrastriatal dopamine transporter and found that the increased dopamine transporter in the amygdala and hippocampus was positively correlated with disease severity. Two studies (Schneier et al., 2000, 2009) from the same team determined the striatal dopamine D_2_ receptor (D_2_R) using different radiotracers while the results were contradictory. They also found that dopamine release had not changed in anxiety patients (Schneier et al., 2009). Another study (Plavén-Sigray et al., 2017) indicated that D_2_R had increased in the orbitofrontal cortex using a high-affinity D_2_R radiotracer whilst a positive correlation was found between disease severity and orbitofrontal cortex D_2_R.

**Table 4:** The results of sensitivity analyses after omitting each study.

**Study omitted**	**SMD**	**Lower limit**	**Upper limit**	***p* value**
**DAT of anxiety disorder**				
Lee et al. (2015)	0.42	−0.96	1.80	0.55
Maron et al. (2010)	−0.26	−1.48	0.96	0.68
Schneier et al. (2009)	0.12	−1.47	1.71	0.88
Tiihonen et al. (1997)	0.47	−0.79	1.74	0.46
Van der Wee et al. (2008)	−0.27	−1.43	0.89	0.65
**DAT of obsessive compulsive disorder**				
Hesse et al. (2005)	0.37	−0.62	1.36	0.46
Hoexter et al. (2013)	0.36	−0.61	1.33	0.46
Kim et al. (2003)	−0.14	−0.98	0.70	0.74
Van der Wee et al. (2004)	−0.09	−1.03	0.86	0.86
**D2R of obsessive compulsive disorder**				
Denys et al. (2004)	−0.64	−1.34	0.07	0.08
Perani et al. (2008)	−0.67	−1.36	0.02	0.06
Schneier et al. (2008)	−0.71	−1.38	−0.05	0.03

*SMD, standard mean difference; DAT, dopamine transporter; D2R, dopamine D2 receptor*.

### Dopaminergic System in OCD Patients

A total of 11 studies examined the dopaminergic system in OCD patients. The mean age of OCD patients ranged from 28 to 40 years old, and the controls were age-sex matched with those patients. Striatal DAT was determined in four studies and all the means were merged together using a meta-analysis (Figure 2B). The overall result showed no significant change and the total pooled SMD was 0.12 (95% CI, −0.69 to 0.94). Although the heterogeneity was extremely high (*I*^2^ = 83%), the result remained stable through sensitivity analyses (Table 4). No significant correlation was claimed by these studies between DAT and disease severity. A research team indicated that the dopamine D_1_ receptor (D_1_R) had decreased in both the striatum and anterior cingulate cortex separately in two articles (Olver et al., 2009, 2010). High negative correlation was found between the total score of the Yale-Brown Obsessive Compulsive Scale (Y-BOCS) and D_1_R in the anterior cingulate cortex but not in the striatum. Four studies (Denys et al., 2004, 2013; Perani et al., 2008; Schneier et al., 2008) determined striatal D_2_R but only three studies with obtainable data could be merged using a meta-analysis (Figure 2C). The merged result was statistically significant and the total SMD was −0.68 (95% CI, −1.23 to −0.12). The heterogeneity was mild (*I*^2^ = 0%) while the result was unstable through sensitivity analyses (Table 4). A study (Denys et al., 2013) that was excluded in the meta-analysis also claimed a decrease of D_2_R. Meanwhile, there was no significant correlation between the Y-BOCS score and D_2_R. In the meantime, one study (Denys et al., 2013) assessed dopamine release in OCD patients and no changes of D_2_R could be found after an injection of amphetamine in the striatum compared with controls. A study (Hsieh et al., 2014) indicated that dopamine synthesis increased throughout the brain, including the left frontal premotor cortex, left posterior cingulate gyrus, left cuneus, left lingual gyrus, right cuneus and precuneus, right lingual gyrus, right middle temporal gyrus, left cerebellum, and right cerebellum.

## Discussion

**Figure 3:**
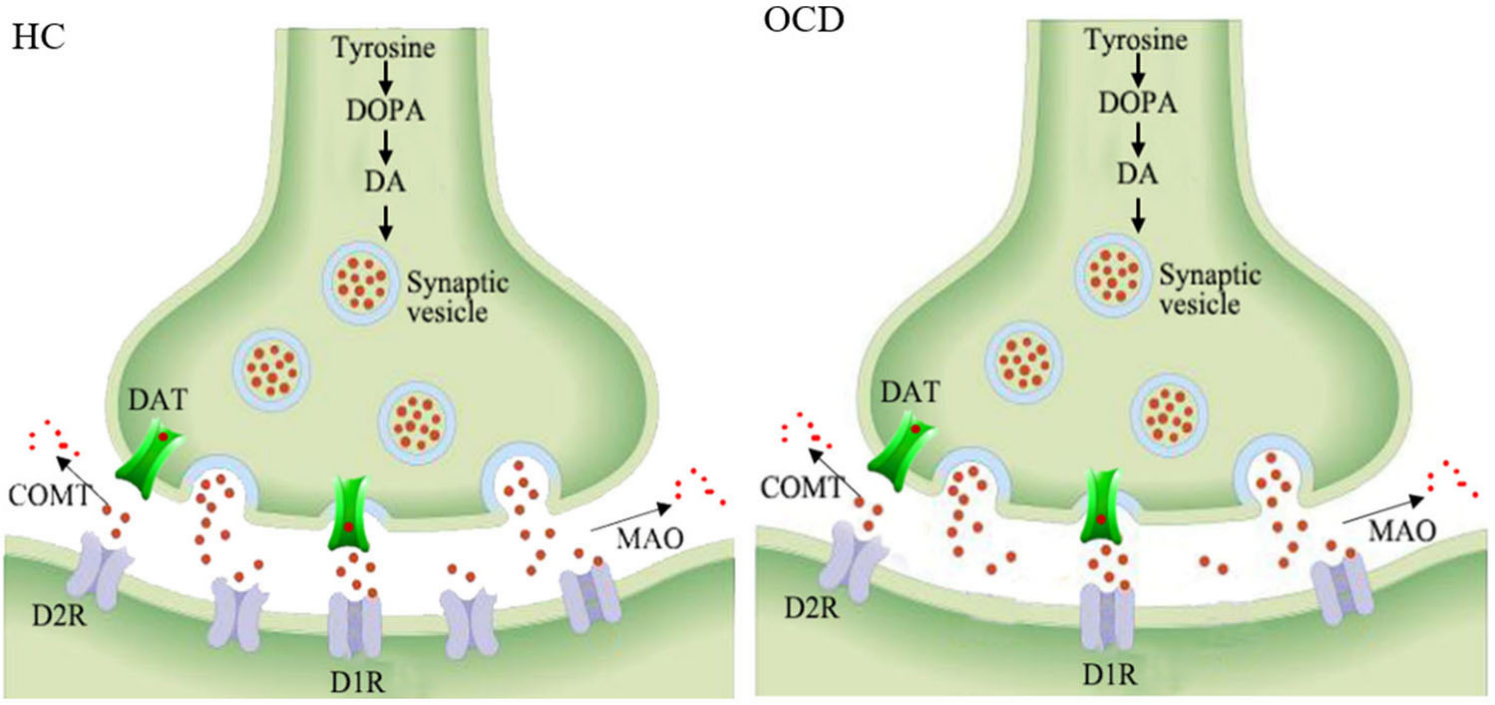
Schematic representation of the decreased striatal D2 receptors in the synapses of OCD patients. HC, healthy control; OCD, obsessive compulsive disorder; DOPA, dioxyphenylalanine; DA, dopamine; DAT, dopamine transporter; COMT, catechol-o-methyltransferase; MAO, monoamine oxidase; D1R, dopamine receptor 1; D2R, dopamine receptor 2.

The dopaminergic system mainly consists of nigrostriatal, mesocortical, mesolimbic, and tuberoinfundibular pathways (Hou et al., 2014). Dopamine is synthesized by dopaminergic neurons in the substantia nigra pars compacta, ventral tegmental area, and arcuate and periventricular nucleus of the hypothalamus (Cortes et al., 2016). After being released into the synaptic cleft, it binds with dopamine receptors 1–5 (mainly D1R and D2R) to exert excitatory or inhibitory signaling. The dopamine in the synaptic cleft can be dissolved by catechol-O-methyltransferase and monoamine, and its level can also be modulated by a dopamine transporter (Figure 3). The involvement of the dopaminergic system in some psychiatric disorders has already been elucidated in detail and might shed light on further drug design strategies (Leggio et al., 2016).

Anxiety and compulsive disorder is among the most common psychiatric disorders, with a lifetime prevalence between 17 and 69% (Kessler et al., 2007). An ongoing issue is that the disorder is under-diagnosed and most patients never receive any treatment. Currently, diagnosis lacks repeatable and objective evidence, and is mainly dependent on the professional skills of psychiatrists and some clinical psychiatric scales. Serotonin reuptake inhibitors and serotonin norepinephrine reuptake inhibitors are usually used as part of the preferred initial treatment (Katzman et al., 2014) with response rates in the range of 30–50% (Reinhold and Rickels, 2015). We have performed a number of studies about this issue before (Dong et al., 2017a,b; Dong et al., 2018a; Chen et al., 2020; Hu et al., 2020). The main aim of this study was to assess whether dopaminergic dysregulation can be detected in anxiety and obsessive disorder with any degree of consistency and specificity. We hope to elucidate the mechanisms of these disorders for clinical diagnosis and further drug development.

Overall, the available studies were quite heterogeneous and often contradictory. Eight studies provided evidence of the dopaminergic system in anxiety patients and eleven studies focused on OCD patients. In the total 19 studies, 10 studies were about DAT, seven studies were about D_2_R, two studies were about D_1_R, two studies were about dopamine release, and only one study was about dopamine synthesis.

The alterations of DAT in patients with anxiety disorders were inconsistent with each other, although the same radiotracer and technique were used. In total, no alteration could be found during the meta-analysis. The primary discrepancy between these studies could have been due to their small sample sizes. Other possible reasons may be the methodological differences. Two studies administered the serotonin reuptake inhibitors citalopram (Tiihonen et al., 1997) or paroxetine (Van der Wee et al., 2008) to block [123I]-β-CIT from binding to serotonin transporters before a SPECT scan while another study did not (Schneier et al., 2009). Reference regions were different as well, including white matter, the cerebellum, and the occipital region. Structural MRI was also used to accurately identify regions of interest in a study (Van der Wee et al., 2008). All of these may partly explain the extremely high heterogeneity of striatal DAT. A recent study from Hjorth using PET (Hjorth et al., 2019) claimed that DAT of the amygdala and hippocampus had increased in 27 anxiety patients. Striatal D_2_R binding significantly decreased in a study using SPECT (Schneier et al., 2000), however, the result had changed when using another radiotracer (Schneier et al., 2009) performed by the same team. The researchers themselves mainly attributed it to the small sample size and not to methodological difference. They also did not find any change in dopamine release after an amphetamine injection. A recent study (Plavén Sigray et al., 2016) using PET with [^11^C]-FLB457 as the radiotracer found D_2_R had increased in the orbitofrontal cortex. However, this finding did not survive after correction for multiple comparisons. In short, no reliable evidence can confirm that the dopaminergic system has participated in anxiety disorders.

There were a total of four studies focusing on DAT in OCD patients and no alteration of DAT was found in OCD patients using a meta-analysis. D_1_R of OCD was determined only in two studies from the same team (Olver et al., 2009, 2010). They found that D_1_R had decreased in the striatum and anterior cingulate cortex. Only seven patients were included in the study so the result needs to be further confirmed. D_2_R had decreased in OCD patients and mild heterogeneity existed. Dopamine release seemed to have no correlation with OCD, either. Only one study (Hsieh et al., 2014) including five patients with OCD determined dopamine synthesis, and it found that dopamine synthesis decreased throughout the brain.

Multiple neurotransmitter systems were involved in the mechanism of OCD according to former studies. It was reported that Glx had decreased in the anterior cingulate cortex while it increased in the caudate using MRS (Brennan et al., 2013; Naaijen et al., 2014). The serotoninergic system had also been significantly altered, as reported in a comparative analysis (Nikolaus et al., 2016). The dopaminergic system may be regulated by the glutamatergic and serotonergic systems (Nikolaus et al., 2010). From this research, we confirmed that striatal D_2_R had decreased in OCD patients. It may be due to receptor internalization, increased levels of endogenous dopamine competing with the radiotracer, or a combination of both (Denys et al., 2013). A dopamine depletion study should be performed to assess whether endogenous dopamine was involved in OCD patients. Currently, serotonin remains the therapeutic target of neurotransmitters and selective serotonin reuptake inhibitors are the first-line drugs to treat OCD patients. However, those drugs can not function well in many patients and induce a number of side effects. Atypical antipsychotics are an important replacement therapy for some OCD patients. These drugs, including risperidone and aripiprazole, can activate D_2_R as well as regulating the serotoninergic system (Stein et al., 2012). Pramipexole, a dopamine D_2_R agonist widely used in PD patients, was also reported to be one of the most important drugs for depression in PD patients. It is worthwhile to explore the usage of these dopamine receptor agonists in OCD patients.

## Conclusion

This is the first systematic review and meta-analysis to elucidate the dopaminergic system in anxiety and compulsive disorder. Studies about this issue are limited in number and contradictory. The most convincing finding is that striatal D_2_R was decreased in OCD patients. Dopamine transporters may have no relationship with anxiety and compulsive disorder. The alteration of the dopaminergic system needs to be further confirmed by more repeatable, reliable, and large-sample size research.

## Data Availability Statement

The original contributions presented in the study are included in the article/supplementary material, further inquiries can be directed to the corresponding author/s.

## Author Contributions

M-XD and LH conceived the study. The data analysis was performed by M-XD, G-HC, and LH. The manuscript was revised by M-XD and LH. All authors contributed to the article and approved the submitted version.

## Conflict of Interest

The authors declare that the research was conducted in the absence of any commercial or financial relationships that could be construed as a potential conflict of interest.
